# Waist Circumference Trajectories in Relation to Blood Pressure and the Risk of Hypertension in Chinese Adults

**DOI:** 10.3390/nu14245260

**Published:** 2022-12-09

**Authors:** Qi Wang, Xiaoyun Song, Shufa Du, Wenwen Du, Chang Su, Jiguo Zhang, Xiaofan Zhang, Bing Zhang, Huijun Wang

**Affiliations:** 1 Key Laboratory of Trace Element Nutrition of National Health Commission of China, National Institute for Nutrition and Health, Chinese Center for Disease Control and Prevention, Beijing 100050, China; 2 Department of Food and School Hygiene, Dalian Centre for Disease Control and Prevention, Dalian 116035, China; 3 Department of Nutrition and Carolina Population Center, University of North Carolina at Chapel Hill, Chapel Hill, NC 27599, USA

**Keywords:** WC, trajectories, blood pressure, hypertension, Chinese adults

## Abstract

Central obesity is associated with a higher risk of hypertension. This study aimed to analyze waist circumference (WC) trajectories and discover their association with blood pressure and the risk of hypertension. The data were obtained from the China Health and Nutrition Survey (CHNS), with a sample of 11,885 adults aged 18 or older. Trajectory groups of WC were identified by group-based trajectory modeling. Three trajectory groups were identified in males: “normal-stable group” (group 1), “normal-increase to central obesity group” (group 2), and “central obesity-slight decrease group” (group 3). There were also three identified in females: “normal-increase to central obesity group” (group 1), “normal-stable group” (group 2), and “central obesity-increase group” (group 3). For males, compared with group 1, systolic blood pressure (SBP) and diastolic blood pressure (DBP) increased by 2.47 mmHg and 2.13 mmHg, respectively, in group 2, and by 3.07 mmHg and 2.54 mmHg, respectively, in group 3. The adjusted hazard ratios (HR) and 95% confidence interval (95% CI) of hypertension in groups 2 and 3 were 1.16 (1.06–1.28) and 1.29 (1.10–1.50), respectively. For females, compared with group 2, SBP and DBP increased by 1.69 mmHg and 1.68 mmHg, respectively, in group 1, and by 4.96 mmHg and 2.77 mmHg, respectively, in group 3. The HR and 95% CI of hypertension in groups 2 and 3 were 1.21 (1.07–1.36) and 1.52(1.17–1.99), respectively. We found that the WC trajectory was a risk factor for hypertension and elevated blood pressure independent of basal WC. Increased risk of hypertension was nonlinearly associated with annual WC increase.

## 1. Introduction

Hypertension is the main cause of premature death worldwide and significantly increases the risks of heart, brain, kidney, and other diseases [1]. According to the World Health Organization, an estimated 1.28 billion adults worldwide had hypertension in 2021 [1]. From 2015 to 2019, the prevalence of hypertension among Chinese adults was 27.5% [2]. Despite the high prevalence of hypertension, awareness, treatment, and control remain extremely low [1], and hypertension remains an insidious disease that is poorly managed among many affected populations [3]. Therefore, the identification and management of the risk factors for hypertension are critical to reducing its public health burden.

Central obesity is an important independent and modifiable risk factor for hypertension [4,5]. WC is frequently used as a marker of central obesity [6]. Previous cross-sectional studies have shown that WC is significantly associated with an increased risk of incident hypertension [4,7,8,9,10]. Prospective studies [11,12,13,14,15] have also shown that increased WC is significantly associated with an increased risk of hypertension. However, these studies focused on one or two WC measurements at specific time points and lacked an assessment of the impact of lifetime WC dynamic patterns on hypertension. Cheng et al. [16] found that sharply increasing trajectories of WC during adolescence were associated with higher risks of incident hypertension in Chinese. Noushin Sadat Ahanchi et al. [17] identified different trajectories for males and females in Tehran and found that both increased WC and highly stable trajectories were associated with a higher risk of incident hypertension. Wang et al. [18] investigated the relationship between the trajectories of WC and cardiovascular disease risk and found that higher WC was associated with an increased risk of myocardial infarction and stroke in four stable trajectory groups in Chinese. To date, few studies have examined the relationship between WC trajectories and hypertension in Chinese adults, so the long-term patterns of WC trajectories in Chinese adults and their association with blood pressure levels remain poorly characterized.

Therefore, the aims of this study were to identify different trajectories of WC and examine their longitudinal association with blood pressure and hypertension risk, and to provide evidence for the prevention or intervention of hypertension and screening of high-risk groups in Chinese adults.

## 2. Materials and Methods

### 2.1. Study Design

This study was based on the China Health and Nutrition Survey (CHNS), a population-based longitudinal survey in China that has been conducted since 1989. Follow-up surveys were conducted in 1991, 1993, 1997, 2000, 2004, 2006, 2009, 2011, 2015, and 2018 to collect anthropometric, clinical, dietary, and other individual, household, and community data. More details were provided in previous articles [19,20]. Our study included eight waves of survey data (CHNS 1993, 1997, 2000, 2004, 2006, 2009, 2015, and 2018) in which WC and blood pressure were measured. The time interval between each two surveys is 4 years, 3 years, 4 years, 2 years, 3 years, 4 years, and 3 years. The Institutional Review Board of the University of North Carolina at Chapel Hill (No. 07-1963), the Institutional Review Committee of the National Institute for Nutrition and Health, and the Chinese Center for Disease Control and Prevention approved the survey protocols, instruments, and procedures for obtaining informed consent (No. 201524). All participants in this study provided written informed consent prior to the surveys.

### 2.2. Study Participants

Between 1991 and 2018, CHNS had 45,340 participants. We excluded 9305 participants younger than 18 years; 411 participants who were pregnant or nursing mothers or with known cardiovascular disease (myocardial infarction, stroke) or cancer; 4123 participants with abnormal data such as body mass index (BMI), WC, SBP, and DBP; 19,222 participants with fewer than 3 measurements of WC; and 394 participants without complete data on all covariates (Figure S1). A total of 59,173 observations from 11,885 participants were included in the group-based trajectory study. The number of visits per participant varied from 3 to 9 with an average of 5 times, including 3 visits, number of participants (*n*) = 3720; 4 visits, *n* = 2081; 5 visits, *n* = 1698; 6 visits, *n* = 1542; 7 visits, *n* = 1335; 8 visits, *n* = 979; and 9 visits, *n* = 530. After excluding participants who had taken antihypertensive medication during the study period because their blood pressure may not reflect the real value, 4493 males and 4773 females were included in the generalized linear analysis. After excluding participants who had hypertension at baseline, 4492 males and 5159 females were included in the Cox proportional hazards analysis.

### 2.3. Measurement of Variables

Data on WC, SBP, and DBP were collected by physical measurements by trained health workers or nurses. At each survey, our trained investigators measured WC in light clothing to the nearest 0.1 cm (cm) using the Seca201 non-elastic tape measure above the participant’s navel while the subject was breathing naturally and upright. According to the Chinese Health Industry Standards (WS/T 428-2013) [21], WC ≥ 90 cm in males and ≥85 cm in females were considered central obesity.

SBP and DBP were measured on the right arm, and the investigators followed the standardized procedures using correctly calibrated mercury sphygmomanometers with cuffs of a certain size. SBP was recorded at the first appearance of the pulse sound (Korotkoff Phase 1) and DBP at the disappearance of the pulse sound (Korotkoff Phase 5). The average of three measurements of SBP and DBP was used for the analysis to reduce the effect of measurement error. According to the 2018 Chinese Hypertension Management Guidelines [22], hypertension was defined as SBP ≥ 140 mm Hg and/or DBP ≥ 90 mm Hg or taking blood-pressure-lowering agents within two weeks or self-reported diagnosis of hypertension.

### 2.4. Measurement of Covariates

Well-trained investigators collected data from participants using standard questionnaires. The following variables were considered covariates: Age, geographic region (urban and rural), educational level, annual household income per capita, survey year, follow-up time, total physical activity (PA), current smoking status, current drinking status, sodium (Na) intake, and potassium (K) intake. In addition, BMI, WC, SBP, and DBP at baseline were included as covariates. Follow-up time was the total time participants stayed from their first survey to their last survey. Baseline covariates were used in the study.

Questionnaires were used to collect respondents’ recalls of PA, including four PA domains, household, occupation, leisure time, and transportation activities. Detailed dietary information was obtained at both the household level and individual level using a weighing method combined with three consecutive 24-h recalls (one weekend and two weekdays). Na (mg/d) and K (mg/d) intake were calculated according to dietary data in the China Food Composition Table.

### 2.5. Statistical Analysis

Characteristics were summarized using the median (IQR) or mean (SD) for continuous variables and counts (proportion) for discrete variables. Males and females have different WC standards, so we analyzed them by gender [21]. WC trajectories were determined using group-based trajectory modeling in the proc traj procedure in SAS 9.4 [23,24]. A censored normal model was used, which was appropriate for continuous outcomes [25]. A model with 2 trajectories was initiated and then 3, 4, and up to 6 trajectory groups were identified. Within each number of trajectory groups, polynomial orders (cubic, quadratic, linear specifications) were tested in order for each trajectory shape until the best-fitting model was established. The best fit depended on model-adequacy criteria [26]. (1) A model with a smaller Bayesian information criterion (BIC) was considered to be a better-fitting model. (2) The average posterior probability of assignment (APPA) value for each group member was determined, where values greater than 0.7 indicated adequate internal reliability. (3) In addition, a value of the odds of correct classification (OCC) within each group was determined, which is recommended to be above 5 per group [27,28].

After testing the normality of residuals and homoscedasticity, a generalized linear model was used to evaluate the association between trajectory groups of WC and blood pressure. The last survey of each participant was used as the outcome.

Cox proportional hazard analysis was used to examine the association between the WC trajectories and the hypertension risk. Entry time was the participant’s baseline age and exit time was the age when the participant was diagnosed with hypertension, censored at the end of the follow-up period, or lost to follow-up, whichever happened first. We calculated HRs and 95% CIs for hypertension.

In an additional analysis, possible exposure–response relationships were explored through a restricted cubic spline [29], and the associations between WC and mean annual increase in WC as a continuous variable with hypertension risk. The cumulative average of WC was calculated as a continuous variable from the time a participant first entered the cohort to the time when he/she was diagnosed with hypertension to better represent the long-term WC levels of this participant [30].

For the generalized linear model analysis and Cox proportional hazards analysis, five models were fitted: Model 1: Unadjusted for covariates; Model 2: Adjusted for age, educational level, geographic region (urban and rural), annual household income per capita, survey year, follow-up time; Model 3: Additionally adjusted for PA, current smoking status, current drinking status, Na intake, K intake; Model 4: Additionally adjusted for baseline BMI, WC; Model 5: Additionally adjusted for SBP and DBP at baseline.

All analyses were performed using SAS 9.4 (SAS Institute, Inc., Cary, NC, USA) and Stata SE15 (Stata Corp., College Station, TX, USA). *p* < 0.05 was considered statistically significant.

## 3. Results

### 3.1. Baseline Characteristics

There were 11,885 individuals in the analysis, including 5686 males and 6199 females (Table 1). The mean (SD) age of all participants was 43.17 (13.91) years. The mean (SD) age for males and females was 42.72 (14.15) and 43.58 (13.66), respectively. The mean (SD) follow-up time of all participants was 14.50 (6.50) years. Most of the participants completed primary school (45.01%), lived in rural areas (65.77%), did not currently smoke (68.42%), and did not currently drink alcohol (63.68%). The initial BMI and WC of all participants were 22.71 (3.22) kg/m^2^ and 78.75 (9.95) cm, respectively. The initial WC of males and females were 80.16 (10.02) cm and 77.45 (9.70) cm, respectively.

### 3.2. Trajectories of WC

**Figure 1:**
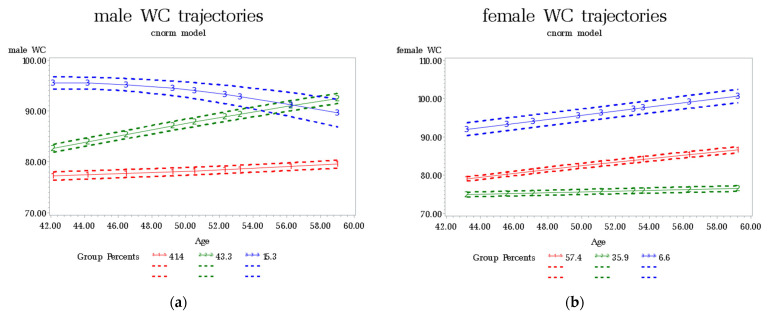
Estimated trajectory groups of WC among males (**a**) and females (**b**). Note: In male, the red line represents the “normal-stable group”, the green line represents the “normal-increase to central obesity group”, the blue line represents the “central obesity- slight decrease group”. In female, the red line represents the “normal- increase to central obesity group”, the green line represents the “normal-stable group”, the blue line represents the “central obesity- increase group”.

Based on model-adequacy criteria, parsimony goals, and interpretability rules, the three-group model was selected for males and females (Figure 1). The model has lower BIC values and ideal APPA values and OCC values. Supplementary Materials Table S1 shows the parameters of the model-adequacy criteria.

The WC trajectories of males and females are shown in Figure 1. For males, three distinct WC trajectories were identified. The red trajectory represents the first group. This group comprised 41.40% of participants, characterized by a low initial WC (77 cm) that remained stable during follow-up. Thus, this group was labelled the “normal-stable group”. The green trajectory represents the second group. The second group comprised 43.30% of participants, characterized by a normal initial WC that continued to increase from 82 cm to over 90 cm during the follow-up. Thus, this group was labelled the “normal-increase to central obesity group”. The blue trajectory represents the third group. The third group comprised 15.30% of participants, characterized by a high initial WC (95 cm) that decreased slightly during follow-up. Thus, this group was labelled the “central obesity- slight decrease group”.

For females, three distinct WC trajectories were identified. The red trajectory represents the first group. The first group comprised 57.40% of participants, characterized by a normal initial WC that continued to increase from 78 cm to 86 cm during the follow-up. Thus, this group was labelled the “normal- increase to central obesity group”. The green trajectory represents the second group. The second group comprised 35.90% of participants, characterized by a low initial WC (75 cm) that remained stable during the follow-up. Thus, this group was labelled the “normal-stable group”. The blue trajectory represents the third group. The third group comprised 6.60% of participants, characterized by a high initial WC (92 cm) that continued to increase. Thus, this group was labelled the “central obesity-increase group”.

### 3.3. Associations between WC Trajectories and Blood Pressure

**Figure 2:**
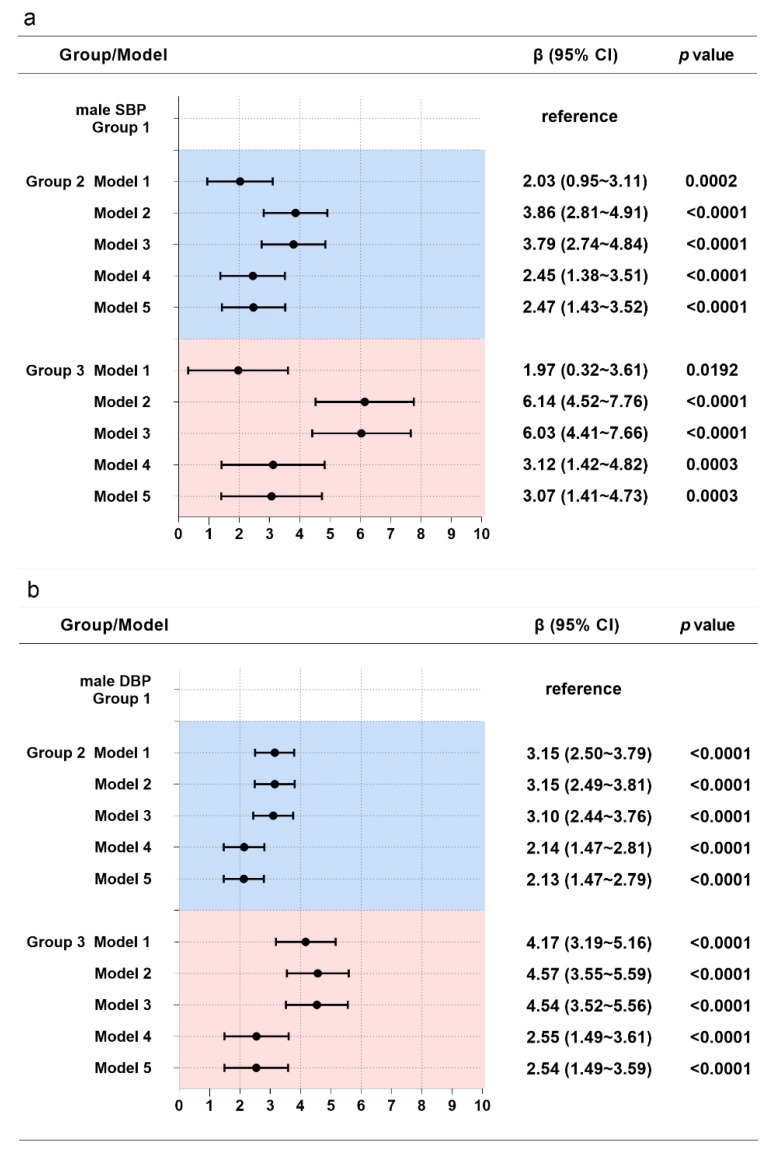
Associations between WC trajectories and SBP (**a**) and DBP (**b**) in males. Note: Model 1 adjusted for no covariates. Model 2 adjusted for age, educational level, geographic region (urban or rural), annual household income per capita, survey year, and follow-up time. Model 3 additionally adjusted for physical activity, current smoking status, current drinking status, Na intake, and K intake. Model 4 additionally adjusted for baseline BMI and WC. Model 5 additionally adjusted for baseline SBP and DBP.

For males, both groups 2 and 3 were found to be associated with increased SBP and DBP compared with group 1 after adjusting for covariates (Figure 2). Participants in group 2 had higher SBP and DBP than those in group 1 by 2.47 mm Hg and 2.13 mm Hg (*p* < 0.05), respectively. Participants in group 3 had higher SBP and DBP than those in group 1 by 3.07 mm Hg and 2.54 mm Hg (*p* < 0.05), respectively.

**Figure 3:**
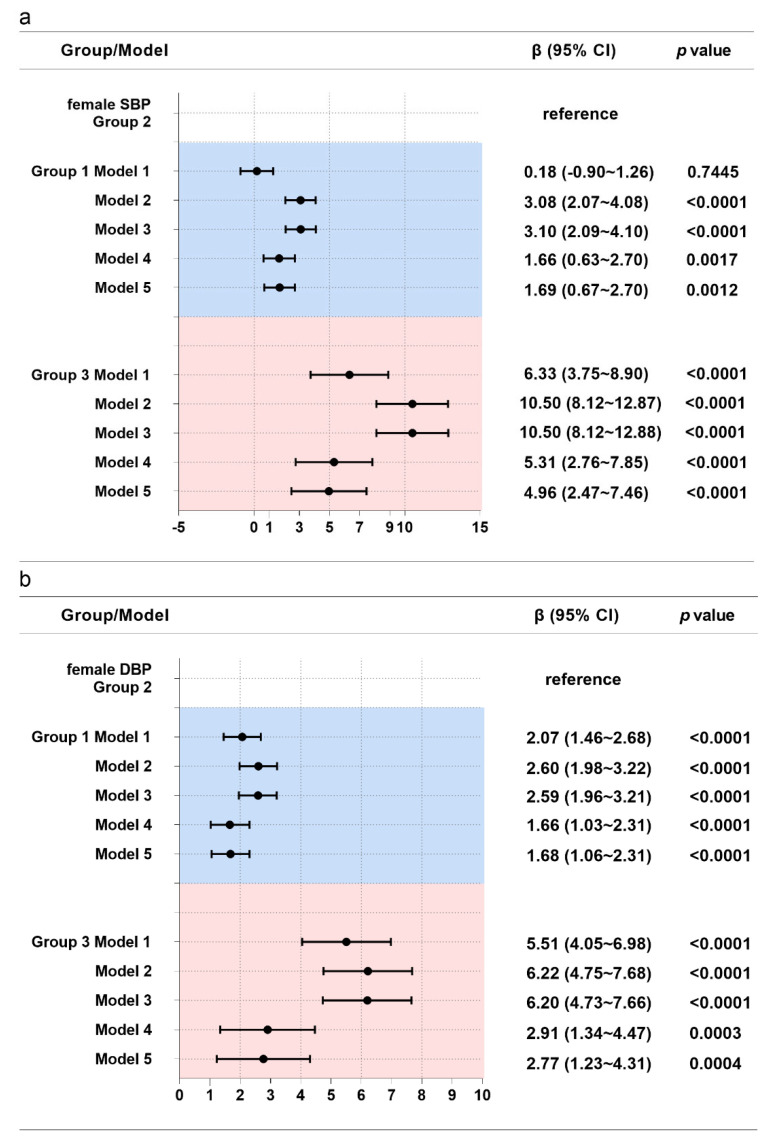
Associations between WC trajectories and SBP (**a**) and DBP (**b**) in females. Note: Model 1 adjusted for no covariates. Model 2 adjusted for age, educational level, geographic region (urban or rural), annual household income per capita, survey year, and follow-up time. Model 3 additionally adjusted for physical activity, current smoking status, current drinking status, Na intake, and K intake. Model 4 additionally adjusted for BMI and WC. Model 5 additionally adjusted for SBP and DBP.

For females (Figure 3), compared with group 2, participants in group 1 had 1.69 mm Hg and 1.68 mm Hg higher SBP and DBP (*p* < 0.05), respectively, and participants in group 3 had 4.96 mm Hg and 2.77 mm Hg higher SBP and DBP (*p* < 0.05), respectively.

### 3.4. Associations between WC Trajectories and Hypertension

Table 2 shows the results of the Cox proportional hazards analysis investigating the association between WC trajectories and hypertension risk in males and females, respectively. For males, compared with group 1, both groups 2 and 3 were associated with a higher risk of hypertension as shown in Table 2 (HR = 1.16, 95% CI = 1.06–1.28, and HR = 1.29, 95% CI = 1.10–1.50, respectively) (*p* < 0.05).

For females, both groups 1 and 3 were associated with an increased risk of hypertension compared with group 2 (HR = 1.21, 95% CI = 1.07–1.36, and HR = 1.52, 95% CI = 1.17–1.99, respectively), (*p* < 0.05).

Table S2 details the associations between covariates and the risk of hypertension. Baseline BMI, WC, SBP, and DBP all increased the risk of hypertension.

### 3.5. Exposure-Response Relationships between WC and Hypertension

**Figure 4:**
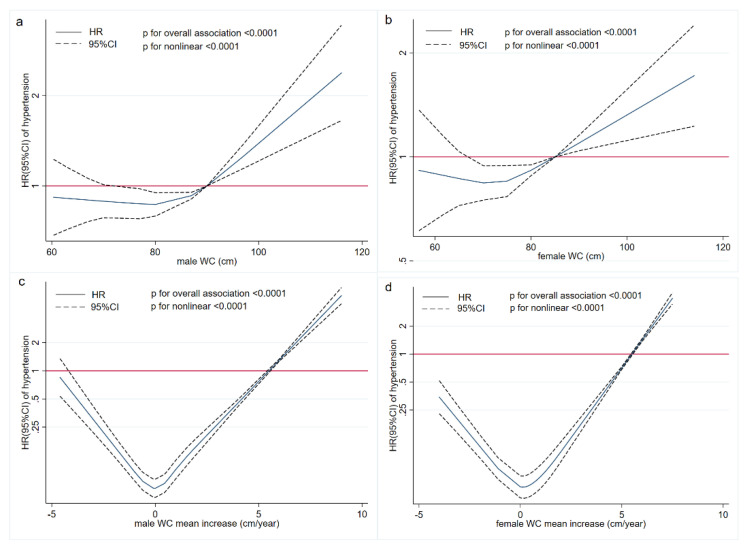
Exposure−response associations of WC and mean annual increase in WC with risk of hypertension by restricted cubic spline for adjusted Cox proportional hazards analysis. (**a**,**b**) the associations of WC with risk of hypertension in males and females, respectively. (**c**,**d**) The associations between mean annual increase in WC and risk of hypertension in males and females, respectively. The red line is the reference line when HR is 1.

Figure 4 presents the exposure–response relationship between WC and hypertension risk. The hypertension risk increased nonlinearly with continuous changes in WC and WC mean annual increase (*p* < 0.0001). These associations were statistically significant when WC > 90 cm in males and WC > 85 cm in females, and with a WC mean annual increase over 5.5 cm in both males and females.

## 4. Discussion

In this Chinese adult cohort study, three distinct WC trajectories in males and females were identified. By using group-based trajectory modeling, we observed different WC trajectory patterns in the population. The findings of this study provide new insights into the prevention and control of hypertension and the identification of individuals at high risk for hypertension and underscore the importance of the WC trajectories for assessing hypertension risk in later life, which is consistent with life-course epidemiological studies [31,32,33].

Several studies on the relationship between WC and the risk of hypertension have been published. Anastase et al. [34] assessed and compared the association of WC with blood pressure (BP) levels and prevalent hypertension in adult Cameroonians. They found that higher WC was associated with a higher risk of hypertension. Other cross-sectional studies found similar results [4,7,8,9,10]. Sun et al. [35] found that every 10 cm increase in WC was associated with an 18% elevated risk of hypertension based on a nationwide cohort Chinese population. Most past studies assessed the association of WC with hypertension risk using WC measured at a single or limited number of time points. There are limited studies on the WC trajectories and their association with blood pressure and hypertension. Therefore, we used latent class trajectory analysis to explore the heterogeneous growth patterns of WC during follow-up, as it is more flexible [26]. We found three distinct trajectory groups in males, namely the “normal-stable group”, “normal-increase to central obesity group”, and “central obesity- slight decrease group”. Unlike males’ WC trajectories, females’ trajectory groups were the “normal-increase to central obesity group”, “normal-stable group”, and “central obesity-increase group”, indicating a higher proportion of adult females than males in the WC increasing trajectory group and a lower proportion of adult females with central obesity than males in the high WC trajectory group. The WC trajectories for males and females were different. The sex-specific WC trajectories in this study differ from other similar studies [16,17,18,36]. Cheng et al. [16] and Wang et al. [18] similarly identified four WC-increasing trajectories in Chinese males and females. Noushin et al. [17] identified WC trajectories among Tehranians and found a higher proportion of females in the WC-increasing trajectory group, which is consistent with our study, but they did not identify a WC-decreasing trajectory in males. Araújo et al. [36] identified three WC trajectories in males and three in females in a Portuguese adult population. Unlike our study, they found high, declining WC trajectories in females. Explanations for gender differences in WC trajectories between males and females may be found by examining the physical and biological makeup of males and females [37,38]. Our study and others did not identify the exact same WC trajectories, which might be attributable to specific differences in study populations.

We found that WC trajectories correlated with blood pressure levels. For males, compared with the normal-stable group, SBP and DBP increased in the normal-increase to central obesity group and increased in the central obesity-slight decrease group. For females, compared with the normal-stable group, SBP and DBP were higher in the normal-increase to central obesity group and higher in the central obesity-increase group. Cross-sectionally examining the association between WC and blood pressure, Fu et al. [39] found that 1 SD higher WC (8.89 cm) in males was associated with a 4.04 mmHg increase in SBP and 1 SD higher WC (8.57 cm) in females was associated with a 3.41 mmHg increase in SBP. Previous studies have examined the relationship between BMI trajectories and blood pressure levels. Fan et al. [40] identified four distinct BMI trajectories in Chinese children: Lean-stable increase, medium-marked increase, heavy marked decrease, and heavy marked increase. They found that individuals in the medium-marked increase group had an average increase of 4.147 mmHg and 2.977 mmHg in SBP and DBP, respectively, and those in the heavy marked increase group had an average increase of 11.660 mmHg and 8.368 mmHg, respectively. There are no comparative studies on the relationship between WC trajectories and blood pressure levels. We are the first to examine the relationship between WC trajectories and blood pressure levels by gender. We used WC trajectories, rather than a single timepoint assessment, to better represent the long-term effect of WC on SBP and DBP.

Few longitudinal studies have investigated the associations between long-term WC trajectories and hypertension risk. Some previous research applied data-driven analytic approaches, such as latent class analysis, to identify distinct and unknown WC patterns in populations and examine their relationships with hypertension or cardiovascular disease (CVD). Wang et al. [18] studied the association between WC trajectories and CVD risk, including stroke and myocardial infarction, in Chinese adults. They identified four stable WC trajectories across all participants: Low stable, moderate stable, moderate-high stable, and high stable. They found that trajectories with elevated WC had a greater risk of CVD events compared with the low stable group. Cheng et al. [16] studied the association between WC trajectories and hypertension risk. They identified four trajectories and found a sharp increase in WC had the highest risk of hypertension. Noushin et al. [17] studied the association between WC trajectories and hypertension risk in Tehran. They found that high-stable and increasing trajectories of WC were associated with a higher risk of hypertension. Similar to these findings, our findings suggest that participants who followed a WC pattern of “normal-increase to central obesity”, “central obesity-slight decrease”, and “central obesity- increase” had a higher risk of developing hypertension later in life. In addition, higher baseline BMI, WC, SBP, and DBP are all risk factors for hypertension.

Notably, a decreasing WC trajectory was identified in males, i.e., WC decreasing from high to low levels. Generalized linear analysis showed that in males, SBP and DBP were 3.07 mmHg and 2.54 mmHg higher in the “central obesity-slight decrease group” than in the “normal-stable group”. Cox regression analyses showed that males in the “central obesity- slight decreasing group” had a 29% higher risk of hypertension than those in the “normal-stable group”. Araújo et al. [36] studied the associations between WC trajectories, BMI trajectories, and cardiometabolic factors in a Portuguese population. They identified a “high, decrease group” in males and found that, compared with the “normal group”, participants in the “high, decrease group” had 3.49 mmHg and 2.25 mmHg higher SBP and DBP, respectively, than those in the “normal group”. Observations from our study and Araújo’s suggest that a WC-decreasing trajectory does not necessarily reduce the risk of hypertension or blood pressure levels. One explanation may be that the initial WC is high, and the reduction is small.

A possible exposure–response relationship, which was rare in previous studies, was also investigated. Results showed a significant association between higher WC and higher risk of hypertension after adjusting for multiple covariates. On a continuum, we found a significantly higher risk of hypertension at WC > 90 cm in males and WC > 85 cm in females. An average annual increase in WC of approximately 5.5 cm was associated with a significantly higher risk of hypertension. Lu et al. [41] examined the relationship between BMI and the waist-to-height ratio (WHtR) as continuous variables and the risk of hypertension and found that the hypertension risk significantly increased when BMI was above 25 kg/m^2^ and WHtR was above 0.5 (the cut-off point was lower in females than in males). These findings are consistent with our study.

The strengths of our study include identifying WC trajectories by using long-term repeated data and latent class trajectory analysis methods, longer follow-up periods, and the inclusion of many potential covariates. However, our study also has some limitations. First, although we adjusted for as many potential covariates as possible, the possibility that other confounding factors were not measured in our study could not be excluded. Second, to meet the requirements of trajectory model fitting, we imposed strict inclusion criteria on our study design, which might reduce the representativeness of the analyzed sample and the generalizability of the study findings. Third, we used a data-driven approach to identify WC trajectories in males and females, and different trajectories were individuated in males and females. This can show trends in WC over time but cannot reflect the specific value of WC change in the population, which is something we should investigate in future studies. Finally, data on 24 h BP, an ambulatory blood pressure measurement (ABPM), can provide more significant value in blood pressure measurement and hypertension diagnosis. However, such data were not yet available in our study.

## 5. Conclusions

In conclusion, the three WC trajectory groups in males and females identified in the Chinese population were associated with different levels of blood pressure and different risks of hypertension. Males in the “normal- increase to central obesity group” and “central obesity- slight decrease group” and females in the “normal- increase to central obesity group” and “central obesity- increase group” had higher blood pressure levels and higher risk of hypertension. The risk of hypertension was significantly higher in males with WC > 90 cm and females with WC > 85 cm. Public health interventions and controls to prevent hypertension should be emphasized in adults, especially for those with WC increasing or stabilizing at high levels. WC > 90 cm in males and WC > 85 cm in females may be the threshold points for developing hypertension. In addition, in the identification of high-risk groups of hypertension, attention should be paid to those with increasing WC.

## Figures and Tables

**Table 1:** Baseline characteristics.

Baseline Characteristics	Male (*n* = 5686)	Female (*n* = 6199)	Overall (*n* = 11,885)
Age, year (mean [SD])	42.72 (14.15)	43.58 (13.66)	43.17 (13.91)
Follow-up time, year (mean [SD])	14.45 (6.39)	14.55 (6.60)	14.50 (6.50)
Education level, *n* (%)			
Primary school and below	2086 (36.69)	3264 (52.65)	5350 (45.01)
Middle school	2028 (35.67)	1665 (26.86)	3693 (31.07)
High school and above	1572 (27.64)	1270 (20.49)	2842 (23.92)
Geographic region, *n* (%)			
Rural	3798 (66.80)	4019 (64.83)	7817 (65.77)
Urban	1888 (33.20)	2180 (35.17)	4068 (34.23)
annual household income per capita, yuan/year (median [IQR])	2292.96 (976.43~5375.76)	2349.88 (962.75~5703.42)	2328.40 (969.98~5558.36)
Physical activity, METs/wk (median [IQR])	169.55 (81.03~315.00)	189.72 (93.88~347.11)	180.33 (86.63~332.30)
Smoking, *n* (%)			
Nonsmoker	2193 (38.57)	5939 (95.81)	8132 (68.42)
Current smoker	3493 (61.43)	260 (4.19)	3753 (31.58)
drinking, *n* (%)			
Nondrinker	2050 (36.05)	5518 (89.01)	7568 (63.68)
Current drinker	3636 (63.95)	681 (10.99)	4317 (36.32)
K intake, mg/d (median [IQR])	1746.27 (1405.66~2144.02)	1535.64 (1232.71~1925.30)	1628.76 (1303.43~2041.04)
Na intake, mg/d (median [IQR])	5805.78 (3806.58~8637.20)	5025.20 (3272.47~7416.11)	5386.81 (3516.91~7981.71)
BMI, kg/m^2^ (mean [SD])	22.56 (3.06)	22.85 (3.36)	22.71 (3.22)
WC, cm (mean [SD])	80.16 (10.02)	77.45 (9.70)	78.75 (9.95)
SBP, mmHg (mean [SD])	120.02 (15.41)	116.22 (17.49)	118.04 (16.64)
DBP, mmHg (mean [SD])	78.39 (10.33)	75.56 (10.74)	76.92 (10.64)

**Table 2:** Associations between WC trajectories and the risk of hypertension by gender.

Gender	Model	Trajectory Groups				
Male		Group 1 (*n* = 1929)	Group 2 (*n* = 2046)		Group 3 (*n* = 517)	
		HR	HR (95% CI)	*p*	HR (95% CI)	*p*
	Model 1	1	1.11 (1.02~1.21)	0.017	1.48 (1.29~1.69)	<0.0001
	Model 2	1	1.38 (1.26~1.51)	<0.0001	1.78 (1.54~2.06)	<0.0001
	Model 3	1	1.37 (1.25~1.50)	<0.0001	1.75 (1.51~2.02)	<0.0001
	Model 4	1	1.16 (1.05~1.27)	0.003	1.26 (1.08~1.47)	0.003
	Model 5	1	1.16 (1.06~1.28)	0.002	1.29 (1.10~1.50)	0.001
Female		Group 2 (*n* = 1548)	Group 1 (*n* = 3398)		Group 3 (*n* = 213)	
		HR	HR (95% CI)	*p*	HR (95% CI)	*p*
	Model 1	1	1.07 (0.98~1.17)	0.137	1.72 (1.42~2.09)	<0.0001
	Model 2	1	1.41 (1.28~1.55)	<0.0001	2.83 (2.31~3.46)	<0.0001
	Model 3	1	1.41 (1.31~1.59)	<0.0001	2.85 (2.33~3.49)	<0.0001
	Model 4	1	1.14 (1.03~1.26)	0.011	1.55 (1.24~1.93)	<0.0001
	Model 5	1	1.14 (1.03~1.26)	0.012	1.47 (1.17~1.84)	0.001

Note: Model 1 adjusted for no covariates. Model 2 adjusted for age, educational level, geographic region (urban or rural), annual household income per capita, survey year, and follow-up time. Model 3 additionally adjusted for physical activity, current smoking status, current drinking status, Na intake, and K intake. Model 4 additionally adjusted for BMI and WC. Model 5 additionally adjusted for SBP and DBP.

## Data Availability

The datasets generated and analyzed during the current study are available from the corresponding author (H.W.) upon reasonable request.

## Supplementary Materials

The following supporting information can be downloaded at: https://www.mdpi.com/article/10.3390/nu14245260/s1, Figure S1: Flowchart of the participants included in the current analysis.; Table S1: Parameters of model-adequacy criteria of the trajectory model; Table S2: Associations between WC trajectories with covariates and the risk of hypertension by gender.
