# Supplementary material for: Waist Circumference Trajectories in Relation to Blood Pressure and the Risk of Hypertension in Chinese Adults
Source: Nutrients. 2022 Dec 9;14(24):5260. doi: 10.3390/nu14245260 (PMC9782435; doi:10.3390/nu14245260)
Supplement: Supplementary file 1 [file nutrients-14-05260-s001.zip › nutrients-2062016-supplementary.pdf]

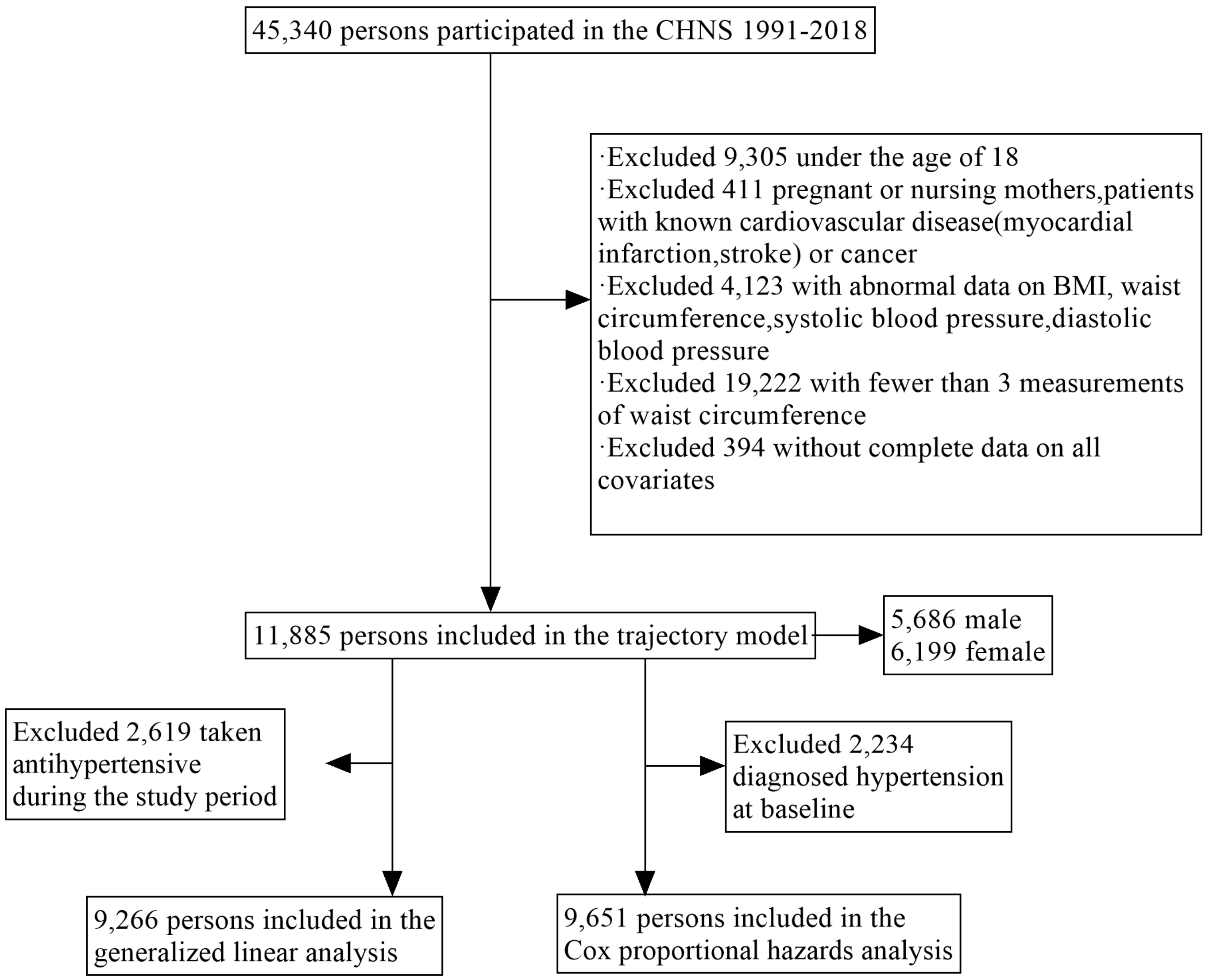

Figure S1 Flowchart of the participants included in the current analysis.

Table S1. Parameters of model-adequacy criteria of the trajectory model

Gender	Trajectory group number	BIC	OCC	APPA	Proportion of individuals in groups (%)
Male	2	-99491.24	13.44/17.43	0.94	54.63/45.37
	3	-96305.18	15.37/8.79/68.79	0.90	41.40/46.39/12.21
	4	-97159.33	31.33/13.58/7.77/105.25	0.86	20.49/32.69/39.17/7.65
	5	-96812.09	7.93/47.23/21.25/9.63/226.74	0.82	33.89/11.84/20.61/29.51/4.15
	6	-96697.35	7.78/22.99/71.40/7.32/13.62/249.08	0.77	28.09/15.41/6.30/27.17/18.99/4.05
Female	2	-111522.30	10.68/15.44	0.93	57.46/42.54
	3	-97891.61	23.22/7.81/41.32	0.88	29.70/53.19/17.12
	4	-109076.20	33.81/11.49/6.03/125.43	0.85	16.36/33.49/43.30/6.86
	5	-108864.50	6.87/6.02/20.89/45.19/171.95	0.79	32.65/34.70/16.92/11.49/4.24
	6	-108709.20	6.67/127.13/12.54/5.23/24.59/210.54	0.74	28.09/4.16/19.52/31.94/12.82/3.47

Note:APPA, average posterior probability of assignment. BIC, Bayesian information criterion. OCC, odds of correct classification.

Table S2. Associations between WC trajectories with covariates and the risk of hypertension by gender

Gender	Male		Female	
Trajectory groups	Model 5 HR and 95%CI	P	Model 5 HR and 95%CI	P
Group 1	reference		1.14(1.03~1.26)	0.012
Group 2	1.16(1.06~1.28)	0.002	reference	
Group 3	1.29(1.10~1.50)	0.001	1.47(1.17~1.84)	0.001
Covariates				
Educational level	0.94(0.89~1.00)	0.056	0.87(0.82~0.94)	<0.0001
Geographic region	0.87(0.78~0.97)	0.012	0.87(0.78~0.97)	0.014
annual household income per capita	0.98(0.94~1.02)	0.334	0.96(0.92~1.00)	0.077
Survey year	0.96(0.95~0.98)	<0.0001	0.97(0.95~0.98)	<0.0001
Physical activity	0.96(0.93~0.99)	0.023	1.00(0.96~1.03)	0.780
Smoking status	1.04(0.95~1.14)	0.372	0.93(0.77~1.13)	0.487
drinking status	1.11(1.02~1.22)	0.019	0.95(0.83~1.08)	0.418
Na intake	1.04(1.01~1.07)	0.014	1.01(0.98~1.04)	0.682
K intake	0.98(0.95~1.02)	0.318	0.98(0.95~1.01)	0.136
BMI	1.12(1.07~1.17)	<0.0001	1.09(1.04~1.14)	<0.0001
WC	1.10(1.05~1.15)	<0.0001	1.09(1.05~1.14)	<0.0001
SBP	1.21(1.16~1.26)	<0.0001	1.23(1.18~1.28)	<0.0001
DBP	1.11(1.07~1.16)	<0.0001	1.10(1.05~1.14)	<0.0001

Age and follow-up time did not satisfy the proportional hazards assumption, so their effects were not estimated here as strata variables.
